# Sphingosylphosphorylcholine inhibits macrophage adhesion to vascular smooth muscle cells

**DOI:** 10.1016/j.bcp.2016.07.004

**Published:** 2016-09-01

**Authors:** Christiane Wirrig, Jenny S. McKean, Heather M. Wilson, Graeme F. Nixon

**Affiliations:** School of Medicine, Medical Sciences & Nutrition, University of Aberdeen, Foresterhill, Aberdeen AB25 2ZD, United Kingdom

**Keywords:** Vascular smooth muscle, Sphingolipids, Macrophage, Nitric oxide synthase, Lipid raft

## Abstract

Inflammation in de-endothelialised arteries contributes to the development of cardiovascular diseases. The process that initiates this inflammatory response is the adhesion of monocytes/macrophages to exposed vascular smooth muscle cells, typically stimulated by cytokines such as tumour necrosis factor-α (TNF). The aim of this study was to determine the effect of the sphingolipid sphingosylphosphorylcholine (SPC) on the interaction of monocytes/macrophages with vascular smooth muscle cells. Rat aortic smooth muscle cells and rat bone marrow-derived macrophages were co-cultured using an in vitro assay following incubation with sphingolipids to assess inter-cellular adhesion. We reveal that SPC inhibits the TNF-induced adhesion of macrophages to smooth muscle cells. This anti-adhesive effect was the result of SPC-induced changes to the smooth muscle cells (but not the macrophages) and was mediated, at least partly, via the sphingosine 1-phosphate receptor subtype 2. Lipid raft domains were also required. Although SPC did not alter expression or membrane distribution of the adhesion proteins intercellular adhesion molecule-1 and vascular cellular adhesion protein-1 in smooth muscle cells, SPC preincubation inhibited the TNF-induced increase in inducible nitric oxide synthase (NOS2) resulting in a subsequent decrease in nitric oxide production. Inhibiting NOS2 activation in smooth muscle cells led to a decrease in the adhesion of macrophages to smooth muscle cells. This study has therefore delineated a novel pathway which can inhibit the interaction between macrophages and vascular smooth muscle cells via SPC-induced repression of NOS2 expression. This mechanism could represent a potential drug target in vascular disease.

## Graphical abstract

**Figure:**
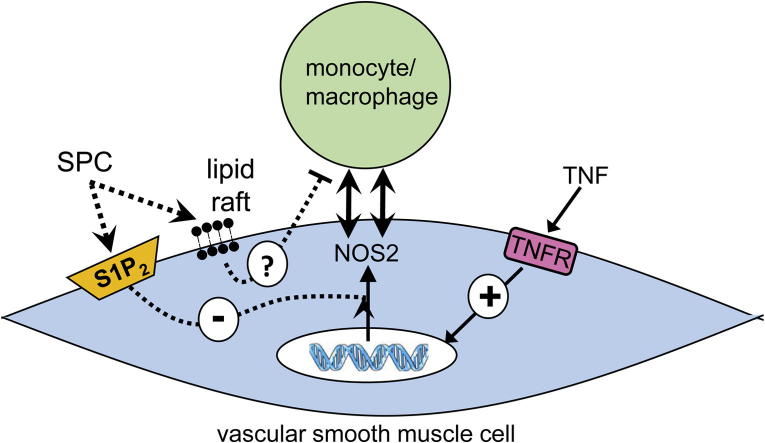

## 1 Introduction

Mononuclear cell adhesion to endothelial cells and their subsequent infiltration into the vascular wall is an important initiating event in the development of atherosclerosis and in blood vessel re-occlusion (restenosis) following angioplasty [1], [2]. Adhesion is typically stimulated by cytokines such as tumour necrosis factor-α (TNF) released from inflammatory cells and vascular smooth muscle cells (SMC) [3]. These cytokines lead to an upregulation of adhesion proteins in endothelial cells, in particular vascular cell adhesion molecule-1 (VCAM-1) and intercellular adhesion molecule-1 (ICAM-1), which facilitate monocyte adhesion [4]. Following adhesion to endothelial cells, tissue infiltration of monocytes and their subsequent differentiation to macrophages lead to a local inflammatory response which drives the development of vascular disease [5]. This consists of changes in the vascular SMC phenotype from a quiescent to a more proliferative and migratory cell type [6]. While research has focussed on the mechanisms of monocyte/macrophage adhesion to endothelial cells, relatively little attention has been paid to interaction of monocyte/macrophage with vascular SMC. Evidence now indicates that monocyte/macrophage adhesion to vascular SMC is likely to be involved in the initiation of vascular disease [7], [8]. It is clear that macrophages and vascular SMC are in direct contact in atherosclerotic plaque regions of human blood vessels [9]. In addition, proliferating neointimal vascular SMC, resulting from vascular injury and endothelial denudation, display an increased binding of monocytes [10]. This evidence demonstrates that monocytes/macrophages can not only adhere to vascular SMC, but that this interaction may also be an integral part of vascular disease pathogenesis.

Sphingolipids are a class of lipid mediators which are naturally occurring in blood and may regulate cell–cell interactions [11]. The most studied sphingolipid, sphingosine 1-phosphate (S1P) is stored in erythrocytes and is an important component of lipoproteins [12], [13]. S1P actions are mediated by a specific class of G-protein-coupled 7-transmembrane receptors consisting of 5 isoforms, S1P_1–5_ [14]. These receptors activate a repertoire of intracellular signalling pathways such as intracellular calcium release and activation of mitogen-activated protein kinases. Most cell types, including vascular SMC, express at least 2 isoforms of the S1P receptor [15], [16]. Recent evidence now suggests that another sphingolipid, sphingosylphosphorylcholine (SPC), can also have important effects on the cardiovascular system [17]. SPC, similar to S1P, is naturally occurring. The source(s) of SPC production, release and metabolism in vivo are not clear although the SPC concentration is raised in serum compared to plasma suggesting release from platelets and SPC (similar to S1P) is also a constituent of lipoproteins [18], [19]. Although a high affinity receptor for SPC has not been identified, SPC can act as a low affinity agonist at S1P receptors [20]. In addition, recent evidence has indicated that SPC signal transduction to intracellular pathways can also occur via lipid raft membrane domains [21], [22]. These lipid raft-dependent effects have been observed in several cell types including vascular smooth muscle and could potentially be receptor-independent [23]. Such effects could involve direct interaction of SPC with these membrane lipid domains. In vascular SMC, we and others have shown that SPC has several actions including vasoconstriction and proliferation [24], [25] although whether these are receptor-dependent or receptor-independent is not known. The effects of SPC on monocyte/macrophage adhesion to vascular SMC have not previously been investigated.

In the current study we have investigated the effect of SPC on macrophage adhesion to vascular SMC using an in vitro co-culture system. SPC significantly decreased the adhesion of rat bone marrow-derived macrophages (BMDM) to rat aortic vascular SMC following stimulation with TNF. This effect was lipid raft-dependent and occurred via an inhibition of NOS2 expression.

## 2 Methods

### 2.1 Preparation of primary cultured cells

Rat BMDM and rat aortic SMC were prepared as previously described [26], [27]. Sprague–Dawley male rats (6 weeks old, 300–350 g) were maintained in accordance with institutional guidelines and were euthanised by approved schedule 1 methods. Bone marrow was extracted from male Sprague–Dawley rats. Rat bone-marrow-derived macrophages (BMDMs) were isolated by aspiration of the femur and tibia and suspended in culture medium comprising Dulbecco’s modified Eagle’s medium supplemented with 10% heat inactivated foetal calf serum, 100 U/ml penicillin and 100 μg/ml streptomycin with the addition of 20% L929-conditioned medium produced using a standard protocol [27]. This yields a population of >95% macrophages after 7 days of culture. Cells were detached by scraping into culture medium or phosphate buffered saline (PBS).

Primary rat aortic SMC were prepared and maintained as previously described [26]. For experimental use cells were grown to 70–90% confluence and placed in serum free DMEM for 24 h before treatment. Primary SMC were used at passage numbers 3–8.

### 2.2 Cell adhesion assay

Rat BMDM were harvested by scraping and washed in PBS. BMDM were labelled by incubating with 1.5 μM calcein acetoxymethylester (AM) for 30 min at 37 °C, centrifuged at 1200 rpm for 4 min and the pellet resuspended in DMEM. BMDM were labelled with calcein AM directly before co-culture. Rat aortic SMC were grown to confluence in 24-well plates. All preincubations of SMC and BMDM were performed separately immediately before co-culture. Rat aortic SMC were washed once with PBS before labelled BMDM were added at a concentration of 5 × 10^6^ ml^−1^. Cells were co-cultured at 37 °C for 15 min. Unbound BMDM were aspirated and wells washed 3 times to remove non-adherent cells. Cells were fixed for 10–15 min at room temperature in 3% paraformaldehyde diluted in PBS. Wells were imaged to measure adherent fluorescent BMDM using a stereo microscope fitted with a super high pressure mercury lamp (SMZ1500; Nikon, Surrey, UK) with excitation and emission wavelengths at 494 nm and 517 nm, respectively. Analysis of the acquired fluorescence images was completed using ImageJ software. Results were expressed as a fold increase compared to control (untreated) samples.

### 2.3 Cell protein extraction and immunoblotting

Cells were incubated with sphingolipids and pharmacological reagents as described [15]. At the end of the incubation period the culture dishes were placed on ice. Culture medium was discarded and cells were rinsed in Tris-buffered saline and scraped into extraction buffer containing 1% Nonidet P-40, 0.5% sodium deoxycholate and 0.1% SDS in Tris-buffered saline, 0.1 mM phenylmethylsulphonyl fluoride and 1 μg/ml pepstatin A. Cell lysates were centrifuged at 14,000 rpm and 4 °C for 10 min. Following measurement of the protein content using a Lowry assay, supernatants were mixed with SDS sample buffer, containing 50 mM Tris–HCl (pH 6.8), 2% SDS, 6% glycerol, 100 mM dl-Dithiothreitol and bromophenol blue. Samples were then boiled for 5 min, cooled on ice and stored at −20 °C until use. For immunoblotting samples were separated by SDS–PAGE and transferred onto nitrocellulose membrane as previously described [26]. These membranes were incubated in primary antibodies which were then followed by detection with HRP-conjugated secondary antibodies and the immunoreactive bands were visualised by enhanced chemiluminescence as previously described [15]. Bands on immunoblots were quantified by densitometry using Biorad Multianalyst software.

### 2.4 Nitrate measurement

The reaction product of nitric oxide (NO), nitrate, was detected using a Griess-based colorimetric assay (Cayman, Ann Arbor, MI, USA). The assay was performed in a 96 well-plate according to the manufacturer’s instructions. Standard dilutions were prepared in duplicate. Conditioned medium from treated rat aortic SMC was loaded in duplicate to triplicate. The colour was allowed to develop for 10 min before measurement of the absorbance at 540 nm wavelength using a microplate reader (Labsystems Multiskan MS, Franklin, MA, USA). Based on the readings of the standard dilutions, a linear standard curve was created and the nitrate concentration in the samples was calculated accordingly.

### 2.5 Preparation of lipid raft fractions

Triton X-100-resistant lipid rafts were prepared previously published [28]. In brief, vascular SMC (2 × 10^7^) were washed with ice-cold PBS and suspended in 1 ml of 2-(morpholino)ethanesulphonic acid (MES)-buffered saline (25 mM MES, pH 6.5, 150 mM NaCl), containing 1% Triton X-100, 10 μg/ml benzamidine, 1 μg/ml leupeptin, 1 μg/ml pepstatin A, 5 mM NaVO_4_, 10 mM NaF and 1 mM PMSF and incubated on ice for 30 min. Extracts were subjected to 10 strokes of a Dounce homogeniser and adjusted to 40% sucrose in MES-buffered saline. Cell extracts in 40% sucrose, were overlaid with 7.0 ml 35% sucrose and 3.0 ml 5% sucrose in MES-buffered saline and centrifuged at 175,000×*g* (Beckman SW41 rotor) for 21 h, 4 °C. Nine fractions (1 ml) were collected from the top of the gradient.

### 2.6 Preparation of sphingolipids and related chemicals

Lyophilised SPC was dissolved in methanol to create stock concentrations of 10 mM SPC. Aliquots of 50 μl were prepared and stored at −20 °C. Before use aliquots were heated at 37 °C for 20 min with brief vortexing every 5 min. The methanol was evaporated with nitrogen gas. Sphingolipids were dissolved in sterile 3.6 mg/ml BSA solution containing 10% DMSO in distilled water. To ensure SPC was dissolved fully, reconstituted aliquots were vortexed for 1 min, followed by heating at 37 °C for 15 min. A concentration of 10 μM SPC was used unless otherwise indicated. These concentrations achieve maximal or close to maximal effects in concentration–effect curves as assessed previously [24].

### 2.7 Analyses and statistics

Each ‘*n*’ number represents a separate primary culture (i.e. a different rat) for both aortic SMC and BMDM. Statistical significance was determined by an unpaired Students’ *t*-test for comparisons between two groups, or analysis of variance followed by a Bonferroni post hoc test for comparisons between more than two groups. A value of *p* < 0.05 was considered significant.

### 2.8 Materials

Sphingolipids including S1P, SPC and 1400W were purchased from Cayman, Ann Arbor, MI, USA. JTE-013 and VPC-23019 were from Tocris, Bristol, UK. Calcein AM was purchased from ANASPEC, Fremont, CA, USA. Antibodies against NOS2, ICAM-1, VCAM-1 and GAPDH were from Santa Cruz Biotechnology, Heidelberg, Germany. Antibodies against IκB-α and phospho-IKKα/β were from Cell Signaling Technology, Danvers, MA, USA. All tissue culture reagents were purchased form Life Technologies, Paisley, UK. All other reagents were purchased from Sigma-Aldrich Company Ltd, Dorset, UK.

## 3 Results

### 3.1 SPC inhibits TNF-induced macrophage adhesion to SMC

**Fig. 1:**
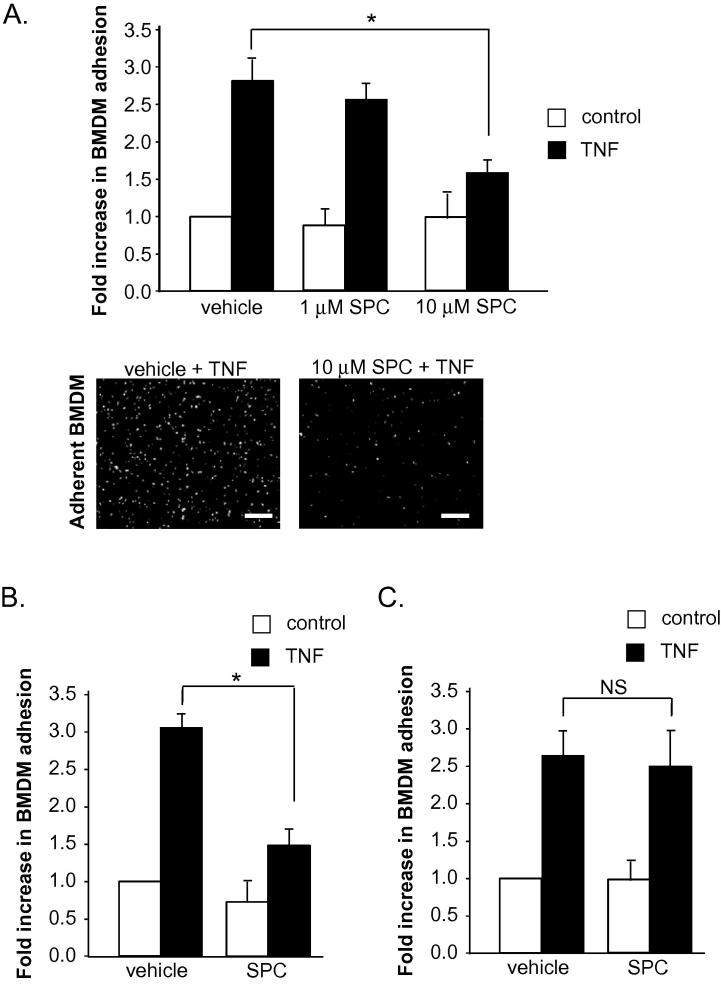
A. Rat aortic SMC and rat BMDM were incubated separately with either 1 μM or 10 μM SPC or vehicle for 24 h in presence (black bars) or absence (white bars) of 10 ng/ml TNF. SMC were subsequently co-cultured with BMDM for 15 min and adherent BMDM counted (*n* = 8). Scale bar = 250 μm. B. Rat aortic SMC were incubated with 10 μM SPC ± 10 ng/ml TNF for 24 h and BMDM were incubated separately with 10 ng/ml TNF only for 24 h (*n* = 12). Rat aortic SMC were subsequently co-cultured with BMDM for 15 min and adherent BMDM counted. C. Rat aortic SMC were incubated with 10 ng/ml TNF for 24 h and BMDM were incubated separately with 10 μM SPC ± 10 ng/ml TNF for 24 h. Adherent BMDM were assessed as above (*n* = 6). Results are presented as fold change of adhesion relative to vehicle treatment. Data are expressed as mean ± sem, ∗ denotes *p* < 0.05, NS denotes not significant.

To assess macrophage adhesion to vascular SMC, we used a co-culture model consisting of a 15 min incubation of primary cultured rat aortic SMC and rat BMDM. In order to produce a pro-inflammatory environment similar to that observed in vivo, we incubated both cell types separately for 24 h with 10 ng/ml TNF before the co-culture incubation. This induced a significant increase in BMDM adhesion to SMC by approximately 3-fold compared to unstimulated cells (Fig. 1A). To initially examine the effects of SPC on BMDM adhesion, both BMDM and SMC were incubated separately with 1 μM or 10 μM SPC for 24 h in the presence of TNF (Fig. 1A). The number of adherent cells following co-culture was unchanged in cells incubated with 1 μM SPC compared to vehicle-treated cells. In contrast, 10 μM SPC preincubation significantly inhibited TNF-induced BMDM adhesion to SMC. SPC preincubation (either 1 μM or 10 μM) without TNF had no effect on cellular adhesion. A concentration of 10 μM SPC was used in all subsequent experiments. As SPC could be acting on BMDM or SMC (or both cell types simultaneously) to produce its anti-adhesive effect, either BMDM or SMC were preincubated with 10 μM SPC for 24 h before co-culture. When SMC were preincubated with SPC and co-cultured with BMDM, SPC retained a significant inhibitory effect on TNF-induced adhesion (Fig. 1B). However, BMDM preincubated with SPC for 24 h and co-cultured with SMC did not alter BMDM adhesion compared to vehicle-treated BMDM (Fig. 1C). This demonstrates that the anti-adhesive action of SPC occurs solely via effects on SMC.

### 3.2 SPC inhibits TNF-induced macrophage adhesion to SMC via S1P_2_ and lipid raft domains

**Fig. 2:**
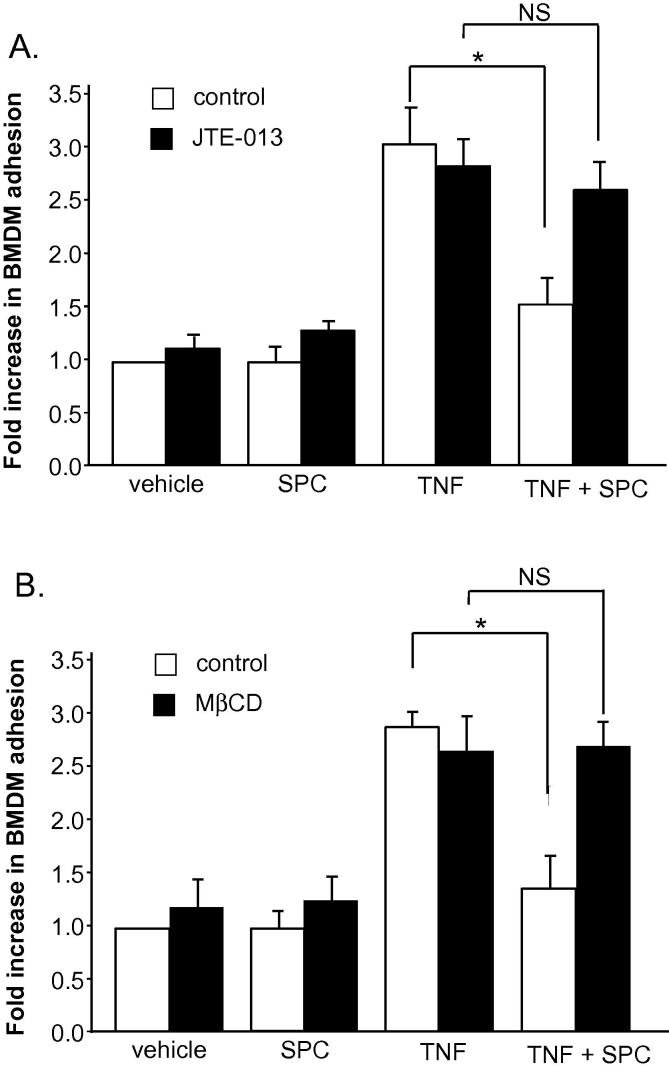
A. Rat aortic SMC were incubated with either 10 ng/ml TNF, 10 μM SPC or TNF + SPC for 24 h in the presence of S1P_2_ receptor antagonist JTE-013 (1 μM added 60 min prior). BMDM were incubated separately with 10 ng/ml TNF only for 24 h. Adherent BMDM were assessed (*n* = 10). B. Rat aortic SMC were incubated with either 10 ng/ml TNF, 10 μM SPC or TNF + SPC for 24 h with 1 mM methyl-β-cyclodextrin (MβCD) added for the final hour of incubation. As above, BMDM were incubated separately with 10 ng/ml TNF for 24 h and following co-culture, adherent BMDM were assessed (*n* = 7). Results are presented as fold change of adhesion relative to vehicle treatment. Data are expressed as mean ± sem, ∗ denotes *p* < 0.05. NS denotes not significant.

We next wanted to determine the potential intracellular mechanisms of the anti-adhesive effect of SPC on vascular SMC. We examined both the potential role of S1P receptors (receptor-dependent) and the possible involvement of lipid rafts (potentially receptor-independent). To determine whether SPC is exerting its effect via S1P receptors, SMC were incubated with the S1P_2_ receptor antagonist JTE-013 (Fig. 2A). Preincubation with JTE-013 significantly prevented the SPC-mediated inhibition of BMDM adhesion to SMC indicate that SPC is acting, at least partly, via the S1P_2_ receptor. In further investigation of S1P receptor subtypes, the experiment was repeated using the S1P_1_/S1P_3_ receptor antagonist VPC-23019 (1 μM and 10 μM). Preincubation with VPC-23019 had no effect on the anti-adhesive action of SPC at either concentration (results not shown). The involvement of lipid rafts was assessed by incubating vascular SMC with methyl-β-cyclodextrin which depletes cholesterol from the cell membrane. We have previously used this protocol in SMC to assess lipid raft involvement in signalling mechanisms [28]. Vascular SMC were incubated with SPC for 24 h and in the final hour of incubation 1 mM methyl-β-cyclodextrin was added. Vascular SMC were subsequently co-cultured with BMDM. While methyl-β-cyclodextrin incubation did not alter levels of cellular adhesion in untreated cells and also did not affect TNF-induced adhesion of BMDM to SMC, the anti-adhesive effect of SPC was abolished (Fig. 2B). This indicates that SPC is also acting at least partly via a lipid raft-dependent mechanism.

### 3.3 SPC-induced changes to cell-cell adhesion occur via regulation of NOS2 expression

**Fig. 3:**
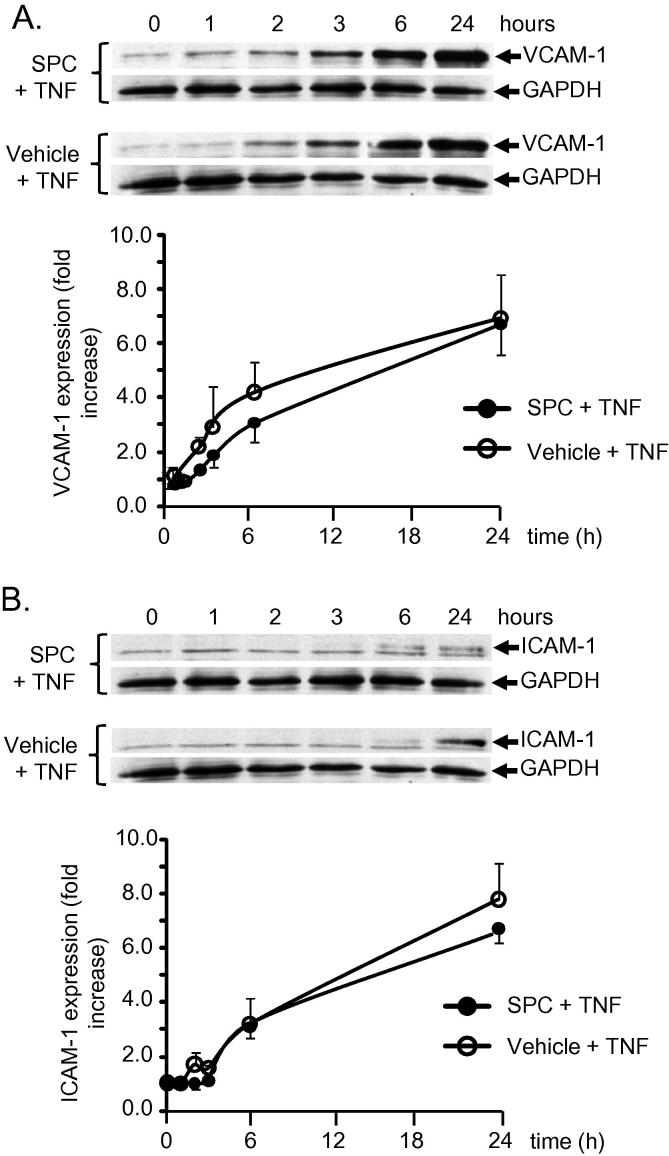
Rat aortic SMC were incubated with 10 ng/ml TNF in presence of 10 μM SPC (black circles) or vehicle (open circles) for times as indicated. Cell protein extracts were subjected to immunoblot analysis for ICAM-1 and VCAM-1 expression. Results are normalised to treatment with vehicle and presented as fold change of VCAM-1 (A) or ICAM-1 (B) protein expression. Representative immunoblots are shown with mean ± sem below, *n* = 12 for each experiment. GAPDH served as a protein loading control.

Increases in cellular adhesion to SMC typically occur via the adhesion proteins VCAM-1 and ICAM-1, therefore the effect of SPC on the expression of these adhesion proteins was assessed. SPC incubation for 24 h had no effect on basal levels of either VCAM-1 or ICAM-1 expression in SMC (data not shown). TNF incubation for up to 24 h produced a time-dependent increase in both VCAM-1 (Fig. 3A) and ICAM-1 expression (Fig. 3B). When SMC were incubated with TNF and SPC, there was no difference in the increase in expression of either VCAM-1 or ICAM-1 compared to TNF incubation alone. Thus, the anti-adhesive effect of SPC does not occur via regulation of VCAM-1 and ICAM-1 expression.

**Fig. 4:**
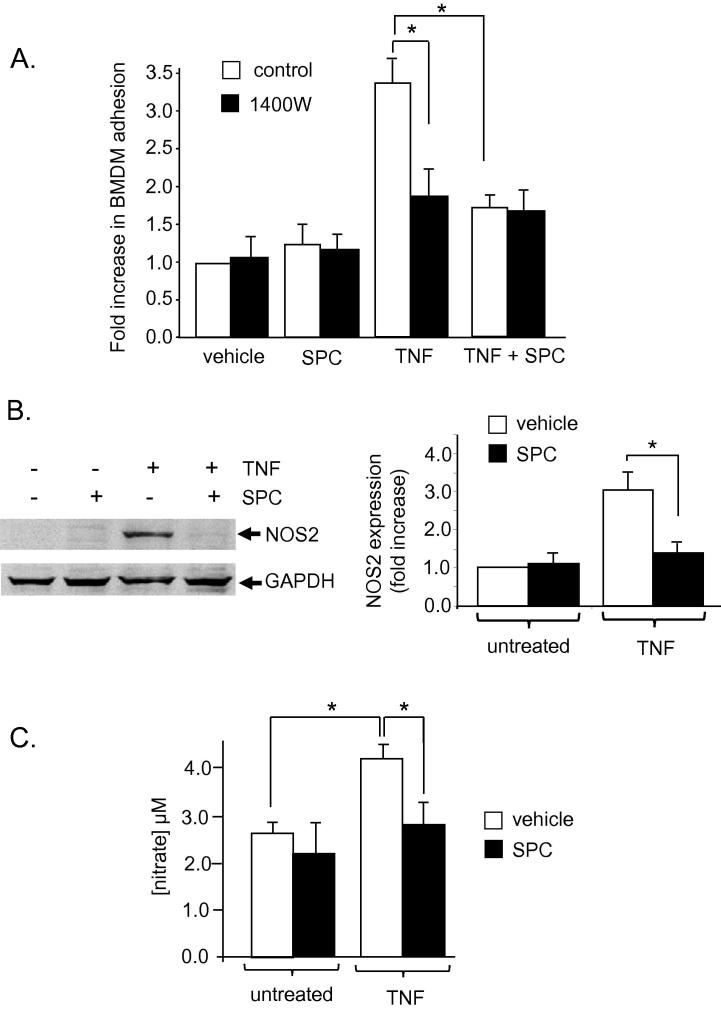
A. Rat aortic SMC were incubated with either 10 ng/ml TNF, 10 μM SPC or TNF + SPC for 24 h in the presence of 1 μM 1400W (a selective NOS2 inhibitor). BMDM were incubated separately with 10 ng/ml TNF only for 24 h. Rat aortic SMC were subsequently co-cultured with BMDM for 15 min and adherent BMDM counted. Results are presented as fold change of adhesion relative to vehicle incubation, *n* = 8. Data are expressed as mean ± sem. B. Rat aortic SMC were incubated as above for 24 h and protein extracts subjected to immunoblotting with anti-NOS2 antibody. Representative immunoblots are shown together with mean data (mean ± sem, *n* = 3). Results are normalised to vehicle treatment and presented as fold change of NOS2 expression. GAPDH served as a protein loading control. C. Rat aortic SMC were incubated as above for 24 h. The nitrate concentration in conditioned media was measured. Data are expressed as mean ± sem, *n* = 4. ∗ denotes *p* < 0.05.

We next investigated other potential signalling mechanisms which may be involved in the SPC-induced decrease in BMDM adhesion to vascular SMC. As nitric oxide synthase (NOS)2 expression (the proinflammatory NOS isoform) is upregulated by TNF in many cell types and its product, NO, has been shown to influence monocyte/macrophage interactions, we next determined if NO had a role in the anti-adhesive effect of SPC [29]. SMC were preincubated with either TNF or SPC or TNF + SPC in the presence of the selective NOS2 inhibitor 1400W. These cells were subsequently co-cultured with TNF-treated BMDM. Inhibition of NOS2 activity significantly decreased the TNF-induced adhesion of SMC to BMDM demonstrating a role for NOS2 in this process (Fig. 4A). The inhibitory effect of 1400W was not additive with the anti-adhesive effect of SPC indicating that SPC and 1400W are acting via the same intracellular mechanisms (Fig. 4A). To examine if SPC can alter the induction of NOS2 expression, SMC were incubated for 24 h with either TNF or SPC or TNF + SPC and NOS2 expression determined. TNF induced a significant increase in NOS2 expression (3-fold compared to control) (Fig. 4B). Incubation with SPC significantly decreased the TNF-induced NOS2 expression to control levels. In order to assess whether this SPC-dependent repression of NOS2 expression resulted in a functional change in NO production, the nitrate concentration in conditioned medium from SMC in Fig. 4B was measured. TNF incubation produced a significant increase in nitrate production. This increase was prevented by co-incubation with SPC (Fig. 4C). SPC therefore decreases NO production by inhibiting NOS2 expression.

### 3.4 SPC prevents the TNF-induced inhibition of NOS2 expression via S1P_2_

**Fig. 5:**
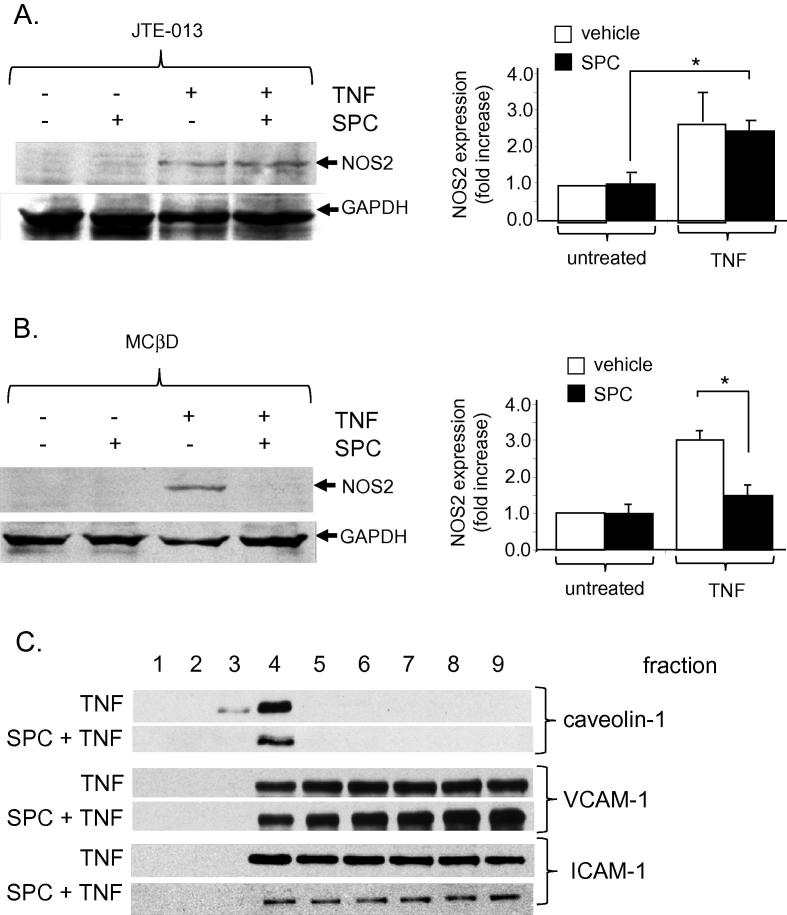
A. Rat aortic SMC were incubated with either 10 ng/ml TNF, 10 μM SPC or TNF + SPC for 24 h with 1 mM methyl-β-cyclodextrin (MβCD) added for the final hour of incubation. Protein extracts were subjected to immunoblotting with anti-NOS2 antibody. Representative immunoblots are shown together with mean data (mean ± sem, *n* = 4, ∗ denotes *p* < 0.05). Results are normalised to vehicle treatment and presented as fold change of NOS2 expression. GAPDH served as a protein loading control. B. Rat aortic SMC were incubated with either 10 ng/ml TNF, 10 μM SPC or TNF + SPC for 24 h in the presence of JTE-013 (1 μM added 60 min prior). Results are normalised to vehicle treatment and presented as fold change of NOS2 expression. GAPDH served as a protein loading control. Data are expressed as mean ± sem, ∗ denotes *p* < 0.05. C. Rat aortic SMC were incubated with either 10 ng/ml TNF or TNF + 10 μM SPC for 24 h and membrane fractions prepared. Extracts were subjected to immunoblotting to reveal expression of caveolin-1 (denotes lipid rafts, predominantly fraction 4), VCAM-1 and ICAM-1. Representative immunoblots are shown, *n* = 4.

The signal transduction mechanisms for the SPC-mediated inhibition of NOS2 expression were investigated. We determined whether this effect was via the S1P_2_ receptor and/or via lipid raft dependent signalling as shown in Fig. 2. Vascular SMC were incubated as above with either TNF or SPC or TNF + SPC for 24 h in the presence of the S1P_2_ antagonist JTE-013 (Fig. 5A). Following JTE-013 incubation, SPC could not decrease NOS2 expression (compared to Fig. 4B) indicating that this effect occurs via activation of the S1P_2_ receptor. To examine whether lipid rafts are involved in the repression of NOS2 expression, vascular SMC were incubated as above with either TNF or SPC or TNF + SPC for 24 h with 1 mM methyl-β-cyclodextrin (to disrupt lipid rafts) added for the final hour (Fig. 5B). Under these conditions which prevent the anti-adhesive effect of SPC (see Fig. 2B), the SPC-induced decrease in NOS2 expression following TNF stimulation was unaffected. This suggests that although SPC can inhibit NOS2 expression via S1P_2_ receptors, this does not require lipid raft domains. The SPC-mediated decrease in BMDM adhesion to vascular SMC must therefore involve 2 distinct mechanisms, one of which is independent of S1P receptor activation and NOS2 but dependent on lipid rafts. In order to further examine this lipid raft-dependent mechanism, we examined the possibility that, although SPC did not alter VCAM-1 and ICAM-1 expression to induce its anti-adhesive effect on BMDM binding, it may regulate the localisation of these proteins in membrane compartments (such as lipid rafts) which can alter adhesion. Evidence indicates that cellular adhesion protein localisation and cell–cell–adhesion can be regulated via lipid rafts [30]. We investigated whether SPC is altering ICAM-1 and VCAM-1 membrane distribution in lipid rafts. This could occur via a direct interaction of SPC with lipid rafts independent of membrane receptors. Vascular SMC were incubated with SPC in the presence of TNF for 24 h. The Triton X100-insoluble extracts containing lipid rafts were separated by sucrose density gradient centrifugation. Collected fractions were subjected to immunoblotting with anti-ICAM-1 or anti-VCAM-1 antibodies. Caveolin-1 was used to identify lipid raft fractions (predominantly located in fraction 4). Following incubation with TNF for 24 h, both ICAM-1 and VCAM-1 were distributed evenly across the membrane fractions including the lipid raft fraction of SMC (Fig. 5C). This was similar to untreated cells (not shown). The addition of SPC to cells for 24 h did not alter this distribution. The anti-adhesive effect of SPC does not therefore alter the membrane distribution of these cellular adhesion molecules.

## 4 Discussion

The interaction of monocytes/macrophages with vascular SMC in vivo is a central component of the pathogenesis of cardiovascular disease [10]. In this study, the effect of the naturally-occurring sphingolipid SPC on macrophage adhesion to vascular SMC was investigated. SPC significantly decreased TNF-induced adhesion of BMDM to vascular SMC in an in vitro co-culture model. This anti-adhesive action of SPC occurred via an effect directly on SMC, but not BMDM. The SPC-induced signal transduction is at least partly receptor-dependent (via activation of the S1P_2_ receptor). The intracellular mechanisms of this effect were not due to changes in the expression or membrane distribution of the adhesion proteins ICAM-1 and VCAM-1, however they were dependent on an inhibition of TNF-induced NOS2 expression via the S1P_2_ receptor. In addition, a distinct lipid raft-dependent mechanism is also involved (independent of S1P receptors). This novel effect of SPC would be expected to decrease localised vascular inflammation and may have important implications for cardiovascular disease.

The adhesion of monocytes/macrophages to vascular SMC can be enhanced through the actions of cytokines such as TNF (as demonstrated in this study), modified low density lipoproteins or growth factors [8]. A major finding of this study is that SPC can alter TNF-induced adhesion of macrophages to smooth muscle cells. This is the first demonstration that SPC has a potentially beneficial effect by inhibiting this specific cell–cell interaction. This study required a relatively high concentration of 10 μM SPC to observe significant effects and it remains to be determined whether local concentrations can be achieved at this level. We have previously shown effects on vascular SMC such as SPC-induced increases in intracellular calcium are maximal at these concentrations [24]. Clearly, for such effects to be observed in vivo, a localised source would be required. SPC is naturally present circulating in blood and is elevated in serum, indicating it is released from platelets and provides potential for significant localised concentration increases [18]. SPC is also a constituent component of lipoproteins, predominantly found in high density lipoprotein (HDL) [17]. It has been suggested that the beneficial effects of sphingolipids in the cardiovascular system, including SPC, may be mediated via sphingolipids bound to HDL [19]. Such an action as that observed in the current study could occur in vivo with HDL as a potential source and scaffold for SPC-induced effects. Regardless of the potential in vivo source of SPC, this study does indicate a mechanism which could be exploited therapeutically as a drug target.

The anti-adhesive action of SPC was mediated entirely via vascular SMC with macrophages having only a passive role. In addition, these effects were mediated, at least in part, via the S1P_2_ receptor on vascular SMC and required functional membrane lipid rafts. Vascular SMC generally express S1P_1–3_ but the relative proportion of each receptor subtype varies in different vascular beds [15]. Although no high affinity receptor for SPC has been characterised, in agreement with our findings suggesting involvement of S1P_2_, a recent study has indicated that SPC can act via S1P_2_ to induce α-smooth muscle actin expression in fibroblasts [31]. Additionally, receptor-independent effects as previously described may also be involved as suggested by studies demonstrating receptor-independent effects including the requirement for lipid rafts in vascular smooth muscle cells [32], [33]. We have similarly demonstrated here that the anti-adhesive effect of SPC requires lipid raft domains although their role is not yet clear. It is possible that SPC, as a sphingolipid, is interacting directly with these cholesterol-rich domains to induce signalling pathways. There is no evidence to suggest that S1P_2_ receptors are resident exclusively in lipid raft domains indicating that the SPC effects we observe here are possibly due to 2 distinct mechanisms: a S1P receptor-dependent pathway and a lipid raft-dependent/S1P receptor-independent mechanism (although notably either blockade of S1P_2_ or disruption of lipid rafts prevented the majority of SPC’s anti-adhesive effects). Further knowledge of SPC pharmacology is required to fully delineate the potentially complex interplay between these mechanisms.

Our investigation of the intracellular mechanisms involved in the anti-adhesive effect of SPC surprisingly did not involve the well characterised cellular adhesion proteins VCAM-1 and ICAM-1. However, we focussed our study on NOS2, the NOS isoform closely associated with inflammation. Cytokines such as TNF have previously been shown to induce expression of NOS2 in vascular SMC [33]. In addition, SMC-monocyte interactions have been observed to increase the expression of NOS2 [29]. We therefore examined the potential role of NOS2 in macrophage adhesion to vascular SMC. TNF incubation increased the expression of NOS2 in vascular SMC. The importance of this increase in NOS2 expression to macrophage adhesion was demonstrated by a selective NOS2 inhibitor 1400W which inhibited TNF-induced BMDM adhesion. This is the first evidence that the adhesion of macrophages to SMC requires the induction of NOS2 and is in contrast to constitutively expressed NOS3 (endothelial NOS) which would decrease monocyte/macrophage binding. The mechanisms involved in the NO-induced increase in adhesion may be related to the high concentrations of NO produced by NOS2 (compared to NOS3) resulting in the formation of peroxynitrite via the actions of free radicals, leading ultimately to nitrosative stress [34]. It has been demonstrated in various vascular disease states (including atherosclerosis and restenosis) that NOS2 expression is upregulated in vascular SMC including those cells found in the neointima [35]. It is therefore clear that in vivo NOS2 is present in a pro-inflammatory environment where monocyte/macrophage recruitment is actively occurring and neointimal SMC are exposed. Having established the importance of NOS2 expression in monocyte/macrophage adhesion, we now reveal that SPC can repress the TNF-induced NOS2 expression in SMC, thereby decreasing adhesion. This occurred via S1P_2_ receptor activation. A previous study has similarly demonstrated that decreasing NOS2 expression can inhibit monocyte adhesion to endothelial cells, although this study did not examine sphingolipid effects [36]. The pathway uncovered by the current study represents a previously unidentified sphingolipid-mediated pathway which extends our knowledge of sphingolipid effects relating to cardiovascular disease.

In conclusion, this study has uncovered a novel pathway which can regulate macrophage adhesion to vascular SMC, a potentially important interaction during the pathogenesis of vascular disease. The naturally occurring sphingolipid SPC inhibits macrophage-SMC adhesion via a mechanism which involves a repression of TNF-induced NOS2 expression in SMC. Such an effect in vivo would be of therapeutic benefit to limit the level inflammation associated with cardiovascular disease and, subject to validation in disease models, could be exploited as a new drug target.

## Disclosures

There are no conflicts of interest.
